# Development and Validation of a Practical Tool for Assessing Acute Pain in the Pediatric Population With Down Syndrome (ANDREAS): A Study Protocol

**DOI:** 10.7759/cureus.87873

**Published:** 2025-07-14

**Authors:** Roberto Latina, Rosaria Gambino, Domenica Matranga, Maria Rita Giammarinaro, Laura Iacorossi, Laura Maniscalco, Noemi Megna, Giustino Varrassi, Martina Busè, Maria Piccione

**Affiliations:** 1 Department of Health Promotion, Mother and Child Care, Internal Medicine and Medical Specialties (PROMISE), Università degli Studi di Palermo, Palermo, ITA; 2 Regional Reference Centre for Congenital Malformations, Chromosomopathies, and Genetic Syndromes, Azienda Ospedaliera Ospedali Riuniti Villa Sofia - Cervello, Palermo, ITA; 3 Department of Nursing, ARNAS Ospedali Civico Di Cristina, Palermo, ITA; 4 Department of Life, Health and Health Professions Sciences, Link Campus University, Rome, ITA; 5 Pain Medicine, Fondazione Paolo Procacci, Rome, ITA

**Keywords:** down syndrome, nursing, pain assessment, pediatric, study protocol, validation

## Abstract

Background

Down syndrome (DS) is one of the most common chromosomal anomalies, affecting a substantial number of newborns globally. Pediatric patients with DS face a range of health challenges, including complex and often underrecognized pain experiences. These children may exhibit atypical responses to pain, such as reduced verbal expression, behavioral changes (e.g., freezing), or lower pain thresholds, that can render conventional pain assessment strategies insufficient. Consequently, there is a critical need for multidimensional tools specifically tailored to the characteristics of this vulnerable population. This study aims to develop and validate a novel mixed-methods instrument - the ANDREAS tool - designed to accurately assess acute pain in children with DS by integrating behavioral observations with self-report measures, where feasible.

Methods

A mixed-methods approach will be employed to adapt a pain assessment scale suitable for children with DS, combining behavioral observations with self-reported measures when possible. Pain-related behaviors will be identified through an extensive literature review and focus group discussions involving researchers, parents, and healthcare professionals. Additional methodological components will include defined randomization procedures for participant selection, clear inclusion and exclusion criteria, a standardized venipuncture protocol to induce pain, and a tailored training program for assessors to ensure methodological rigor. The resulting instrument, ANDREAS, will undergo validation using exploratory factor analysis and internal consistency testing (Cronbach’s alpha).

Results

The study is expected to yield a validated tool capable of capturing both the presence and severity of acute pain in pediatric patients with DS. Key behavioral indicators, such as freezing, defined as the absence of movement for at least five seconds during a painful stimulus, will be operationalized and assessed using a structured 0-4 rating scale. The integration of behavioral and self-report data is anticipated to enhance the sensitivity and specificity of pain detection in this population.

Conclusions

The ANDREAS instrument is expected to provide a reliable, valid, and clinically meaningful method for assessing acute pain in children with DS. By combining behavioral observations with self-report, it introduces a novel, multidimensional approach that may improve the accuracy of pain evaluation and inform more effective clinical management strategies. Future research will focus on further validating the instrument’s psychometric properties and exploring its use across diverse clinical settings.

## Introduction

Down syndrome (DS) is the most common chromosomal anomaly [1], affecting approximately one in every 640 newborns [2]. It is characterized by the presence of an extra chromosome, which leads to atypical psychophysical development and associated disabilities. Individuals with DS often face numerous health complications due to multiple comorbidities, frequently requiring medical interventions, surgical procedures, and hospitalizations. As a result, they experience pain more often than their typically developing peers [3].

Children with DS are known to have a lower pain perception threshold [4], reduced pain tolerance, delayed reaction times, prolonged pain experiences, and altered nociceptive signal conduction and modulation [5,6]. Moreover, their expression of pain is often ambiguous and challenging to interpret, both qualitatively and quantitatively [7]. Verbal responses and crying may be minimal or absent, while facial expressions tend to remain evident [8,9], sometimes accompanied by immobility of the face and body. This response pattern, known as “freezing,” refers to a paralytic reaction that may give the false impression of pain insensitivity.

Recent studies focusing on facial expressions provide promising insights into understanding pain in this population. Careful observation of facial cues may help clinicians detect pain without being influenced by the patient’s cognitive abilities. Additionally, the pain experience in individuals with DS can vary greatly due to heterogeneous structural and functional alterations in the central nervous system, which affect the transduction, transmission, modulation, and perception of sensory information [10].

Pain recognition is further complicated by subtle or idiosyncratic behaviors and wide individual variability in physical and expressive abilities, which are closely tied to different levels of cognitive development. Although self-report remains the gold standard for pain assessment, it is often difficult to apply and interpret in individuals with intellectual disabilities [5]. Children with such disabilities face considerable disparities in pain-related care and remain significantly underrepresented in pain research. Standard pain assessment tools are frequently inadequate for this population, especially for those who are nonverbal or unable to understand and follow instructions [11].

Importantly, children with DS are not insensitive to pain; rather, they tend to express discomfort more slowly and less clearly than their typically developing peers. This underscores the importance of implementing proactive pain management strategies, even in the absence of obvious pain cues. Despite this, current research appears insufficient in addressing the healthcare needs of this vulnerable group [12].

Therefore, it is a critical priority to develop assessment tools specifically designed to evaluate pain in children with DS and other forms of intellectual disability. The primary aim of this study protocol is to develop and validate a psychometrically robust, mixed-method instrument - ANDREAS - capable of accurately detecting and measuring the presence and intensity of pain in pediatric patients with DS. A secondary aim is to identify behavioral indicators of pain in relation to varying degrees of intellectual impairment.

## Materials and methods

Research hypothesis

Current pain assessment tools are not sufficiently specific, valid, or reliable for accurately measuring pain in children with DS.

Study objectives

The primary objective of this study protocol is to design and validate a reliable mixed-method assessment tool that incorporates both self-report and hetero-evaluation by healthcare professionals and family caregivers to accurately detect and measure pain in the pediatric population with DS. A secondary objective is to identify behaviors specifically indicative of pain in children with DS, according to their degree of intellectual disability.

Study design

This study adopts a single-center, observational, mixed-method design that integrates both qualitative and quantitative methodologies. The qualitative phase will involve a focus group with parents of children and adolescents with DS [13]. Semi-structured interviews, consisting of seven ad hoc questions (Table 1), will be audio-recorded, transcribed verbatim, and analyzed thematically. All qualitative procedures will adhere to the Consolidated Criteria for Reporting Qualitative Research (COREQ) [14].

**Table 1:** Questions for the qualitative research DS, Down syndrome

Questions for a qualitative study addressed to parents of children with DS
Experience: Can you describe a specific time or situation when you recognized that your child was experiencing pain?
Facilitating factors: What signs or behaviors have helped you understand that your child was in pain? (How does your child communicate pain? Does your child use words, gestures, or other forms of expression? What kinds of verbal or nonverbal signs do you observe when your child is in pain?)
Hindering factors: What challenges have you faced in recognizing pain in your child? (Do you believe there are behaviors typical of DS syndrome that complicate pain recognition?)
Experience with healthcare professionals: What aspects of communication with healthcare professionals have you found most helpful or least helpful regarding your child’s pain?
Suggestion: Is there anything else you would like to share regarding your child’s experience of pain that we have not discussed so far? What advice would you offer to other parents who are facing similar situations?
Facilitating factors: What factors do you believe contribute to reducing your child’s perception of pain? (What elements help make the experience less traumatic?)
Relational question: How would you describe your emotional reactions as a parent when coping with your child’s pain?

Study procedures

The study will be conducted in five distinct phases. The first phase will focus on the development of an adapted “faces” pain assessment tool specifically designed for pediatric patients with DS. A body diagram for pain localization may also be incorporated. The tool will retain a self-report function to capture the pain experience directly from the young patients.

In the second phase, pain-related behaviors will be identified through structured interviews with professional caregivers (e.g., nurses, physicians, and psychologists). Caregivers will assess 12 predefined behaviors, drawn from established observational tools (e.g., brow lowering, eye squeezing, nasolabial furrow, and finger splaying), indicating whether they have observed each behavior during venipuncture procedures in children with DS. Each behavior will be marked as “observed” or “not observed.” Simultaneously, caregivers will complete an adapted six-face pain scale tailored for pediatric patients with DS, with two illustration sets (one featuring a child with glasses and one without) to accommodate visual aid preferences. The face that best represents the child’s pain intensity at the time of venipuncture will be selected. Data collected during this phase will contribute to refining the behavioral indicators included in the ANDREAS tool.

The third phase will further explore pain-related behaviors through a focus group involving parents. Using qualitative methods, parents will provide insights into their children’s pain expressions, offering a deeper understanding of individual variability.

Based on input from healthcare professionals and parents, the fourth phase will involve finalizing the list of items and measurement scales for the ANDREAS instrument. The tool will then undergo validation, with particular focus on its psychometric properties. An observational study will assess face, content, and construct validity through exploratory factor analysis (EFA), as well as evaluate reliability.

In the fifth and final phase, a comprehensive report will be prepared, and the results will be disseminated through publication. These findings aim to improve pain assessment practices for children with DS, enabling more accurate and individualized clinical interventions.

Each study phase involves specific procedures and participant groups, selected according to the phase’s objectives. Inclusion and exclusion criteria will be defined separately for the qualitative and quantitative components. The expected study duration is approximately 18 months, covering recruitment, data collection, and validation.

Qualitative study sample

The qualitative sample will consist of approximately 30 parents of children and adolescents with DS, chosen to reflect a range of ages and comorbidities while maintaining comparable levels of cognitive impairment. This sample size is based on evidence that 99.3% of concepts, themes, and content typically emerge after about 30 interviews [15]. Recruitment will continue until data saturation is reached [16].

Inclusion criteria include parents of children or adolescents with DS who present with mild to moderate-severe cognitive impairment, regardless of clinical condition. Participants must provide written informed consent and agree to participate in focus groups.

Exclusion criteria include parents who are unable to actively participate due to medical or psychological conditions, those whose children do not consent to the study, or those whose children’s cognitive impairments and pain-related behaviors are too complex to assess reliably.

Quantitative study sample and eligibility criteria

The sample size for the observational study is based on a hypothesized prevalence of pain in the pediatric DS population of approximately 30% [10], with a 95% confidence level and a margin of error of 7%. The standard formula for estimating sample size in prevalence studies is as follows:

\[n = \frac{Z^2 \cdot p \cdot (1 - p)}{d^2},\]

where Z = 1.96 (for 95% confidence), p = 0.30, and d = 0.07.

The resulting estimated sample size is approximately 165 child participants. All pediatric subjects whose parents provide informed consent will be eligible for inclusion.

Inclusion Criteria

Eligible participants will be children older than eight years whose parents voluntarily agree to enroll them in the study. This cohort will include children whose parents participate in the initial focus group as well as those involved in testing the newly developed pain assessment tool. Instrument testing will take place in healthcare facilities during venipuncture procedures in order to evaluate the tool’s capacity to detect the presence and intensity of pain.

Exclusion Criteria

Children will be excluded if their parents do not provide, or are unable to provide, informed consent. Additionally, any child with profound cognitive impairments that preclude reliable pain assessment will be excluded.

Variables to be collected

Audio recorders will be used to capture the focus group discussions and interviews. A specifically developed pain measurement tool will be employed to convert parental reports into coded data for further analysis.

Parent-related variables will include the parents’ age, sex, and educational background, as well as the number of years they have spent as caregivers or in direct care of their child. Information will also be gathered on the presence of other children in the family, the number of hospitalizations the child has undergone, and the reasons for these hospitalizations. Parents will be asked to describe their observations of their child’s experiences during pain episodes, including any distinctive behaviors exhibited during such episodes. Additionally, the impact of the child’s pain on family life, education, and learning will be documented.

Child-related variables will encompass the child’s age (restricted to those older than 8 years), gender, and current school grade. The child’s level of cognitive development will be assessed using the Leiter International Performance Scale, Third Edition (Leiter-3) [17]. The Leiter-3 is a standardized instrument designed to measure intelligence quotient (IQ) and cognitive ability in individuals aged three to over 75 years. Unlike conventional IQ tests, it focuses on fluid, nonverbal reasoning skills, making it especially suitable for individuals with cognitive or communication difficulties, including those with brain injuries or neurodegenerative conditions such as Alzheimer’s disease, Parkinson’s disease, or dementia.

Operationalization of the ANDREAS tool items

The ANDREAS tool will consist of observational items designed to capture specific behavioral indicators of pain in children with DS. Each item will follow a standardized structure and will be rated using a 5-point Likert scale (0 = never, 4 = always), based on either direct observation or caregiver report. Table 2 provides illustrative examples of item structure, behavioral domains, and the scoring criteria applied in the ANDREAS tool. The items will be organized into thematic domains such as facial expression, vocal behavior, and motor response. Their formulation will be grounded in qualitative findings derived from interviews and focus groups. To ensure consistent administration and interpretation, a coding manual containing operational definitions and detailed scoring guidelines will accompany the tool.

**Table 2:** Examples of observational items included in the ANDREAS tool

Item example	Behavioral domain	Operational definition	Scoring scale
The child vocalizes through crying, moaning, or atypical sounds not attributable to known communicative patterns.	Vocal expression	Nonpurposeful vocalizations that occur in conjunction with observable signs of distress.	0 = never; 1 = rarely; 2 = sometimes; 3 = often; 4 = always
The child suddenly becomes immobile or assumes a rigid posture during a painful episode.	Motor behavior	A sudden cessation of voluntary movement lasting at least five seconds, not explained by external environmental stimuli.	0 = never; 1 = rarely; 2 = sometimes; 3 = often; 4 = always

Item development will follow an iterative, expert-led process involving multiple rounds of refinement, peer review, and pilot testing. This approach is intended to ensure that the tool remains closely aligned with the theoretical framework of pediatric pain in children with DS while also minimizing the risk of construct drift or item overfitting during the scale development process.

Training and standardization procedures

Prior to data collection, all researchers and healthcare professionals responsible for administering the ANDREAS tool will participate in a standardized training program. This program will include theoretical instruction, practical simulations using case studies, and the review of annotated video recordings. Training will focus on ensuring consistent interpretation of items, accurate scoring, and reliable behavioral observation techniques. Inter-rater reliability will be evaluated during pilot testing by calculating agreement levels using Cohen’s kappa coefficient.

Behavioral coding protocol

Each behavioral indicator included in the ANDREAS tool will be operationally defined within a structured coding manual. This manual will outline detailed criteria for identifying and rating observable pain-related behaviors. For example, “freezing” will be defined as the sudden cessation of voluntary movement lasting at least five seconds, unrelated to any external stimulus. Depending on the item format, each behavior will be rated either using a binary presence/absence code or a 5-point Likert scale (0 = never, 4 = always). All data will be initially recorded on paper forms and then transcribed into a secure, GDPR-compliant digital platform for managing sensitive health information. Specific coding protocols will be uniformly applied across all cases to ensure the accuracy, reproducibility, and traceability of behavioral observations.

Statistical analysis

Descriptive statistics will be used to characterize the study population, including measures such as means, standard deviations, medians, interquartile ranges, proportions, and correlation coefficients. Summary tables will present key sociodemographic and clinical variables, including sex, age, pain localization, duration, and intensity, along with their respective ranges.

Psychometric validation of the ANDREAS instrument will proceed in multiple stages. Construct validity will be assessed through EFA, depending on data characteristics and sample size. Prior to EFA, the suitability of the dataset will be determined using the Kaiser-Meyer-Olkin measure of sampling adequacy, with a threshold of ≥ 0.60, and Bartlett’s test of sphericity, with statistical significance set at p < 0.05. Factor extraction will be based on eigenvalues greater than 1 and scree plot inspection, with factor loadings of ≥ 0.40 considered acceptable.

Internal consistency will be evaluated using Cronbach’s alpha, with coefficients of ≥ 0.70 interpreted as satisfactory. Additional confirmatory analyses may be conducted depending on the robustness of the data. All statistical analyses will be performed using appropriate and validated software tools.

Ethical considerations

In accordance with the Guidelines for the Classification and Conduct of Research Studies, all data will be processed in compliance with current Italian legislation (Art. 13 of Law 196/2003; D.M. 5 July 1997 guidelines; Legislative Decree 200/2007), as well as the ethical principles of the Declaration of Helsinki. Data collection will involve both parental interviews, following informed consent obtained from parents for themselves and their children, and observational methods involving the children. Anonymity and confidentiality will be strictly upheld, with all personal data handled according to the provisions of Italian privacy laws (DL 196/03). A confidential list linking participant identification codes to their identities will be maintained by the project coordinators at the Department of Health Promotion, Mother and Child Care, Internal Medicine and Medical Specialties (PROMISE), Università degli Studi di Palermo. The study protocol received approval from the Palermo 3 Ethics Committee (protocol number 163_03/04/2024).

## Results

The study is expected to identify a range of behavioral indicators associated with acute pain in children with DS, as recognized by both professional caregivers (e.g., clinicians) and nonprofessional caregivers (e.g., parents). These indicators, along with an adapted six-face pain scale specifically tailored for the pediatric DS population, will support the interpretation of behavioral expressions that may be indicative of pain in children with mild to moderate cognitive impairment. Psychometric validation of the ANDREAS instrument is anticipated to confirm its reliability and validity, thereby minimizing measurement errors and reducing the risk of inaccurate pain assessment. Enhanced accuracy in pain evaluation will support the timely implementation of appropriate pharmacological and nonpharmacological interventions. The tool will be designed for practical use in both clinical and home settings by healthcare professionals and family caregivers alike.

## Discussion

Nurses, physicians, and other healthcare professionals play a vital role in advocating for patients who are unable to clearly communicate their pain experiences [5]. This study aims to develop and validate a novel mixed-methods instrument (ANDREAS) that integrates self-reporting with observational behavioral assessment to accurately evaluate acute pain in pediatric patients with DS. These children frequently experience both acute and chronic pain [3]. Despite this, individuals with cognitive disabilities continue to face substantial inequities in pain-related care, and research specifically involving this population remains limited. Many individuals with disabilities, especially those who are nonverbal, are unable to self-report pain. Moreover, children and adolescents with cognitive impairment may be unable to provide informed consent or assent for research participation, and their dissent may go unnoticed [11].

In light of this complex clinical context, the present study will focus on pediatric patients with mild to moderate cognitive impairment, emphasizing the critical collaboration between parents and dedicated healthcare professionals. Further exploration of the mechanisms underlying altered pain perception, attributable to structural and functional brain differences, is necessary to improve pain management strategies in patients with DS [10]. For these children, the choice of healthcare provider is often highly personal; they tend to gravitate toward the nurse, doctor, or psychologist they trust most. Given the heterogeneous cognitive profiles within the DS population [5], the capacity to verbalize pain varies significantly. Many parents, acting as proxy reporters, note that recognizing pain behaviors in their children is a complex and highly individualized task, evolving over time with the child’s development [18].

Proxy reporters may include parents, family members, unlicensed caregivers, and professional healthcare workers. Valuable and credible information can often be obtained from those who are most familiar with the child’s typical behaviors and responses [5]. A valid and reliable pain assessment is fundamental to ensuring a safe, patient-centered approach to analgesia, one that is tailored to each individual’s needs and prevents both underestimation and overestimation of perceived pain. At the same time, care must be taken to minimize possible complications [19].

To achieve truly personalized care for children with DS, the ANDREAS tool must demonstrate strong psychometric properties, including high reliability and validity. Using tools that are both reliable and validated ensures that clinicians base their pain assessments and treatment decisions on appropriate and evidence-based criteria. It is important to emphasize that behavioral pain scores are not equivalent to self-reported numerical pain ratings. Rather, behavioral tools should support healthcare providers in identifying the presence of pain, monitoring changes over time, and evaluating the effectiveness of pharmacological and integrative treatments [5].

Although pain is the most common health issue worldwide, affecting all populations regardless of background, individuals with mild to moderate cognitive impairment continue to face barriers in expressing their needs. As such, healthcare systems must guarantee equitable access to effective pain management for these vulnerable groups. While this protocol introduces a clinically meaningful and innovative tool, certain methodological and contextual considerations should be acknowledged.

This study will be conducted at a single center and will focus on a specific subgroup of children with DS, which may limit the generalizability of its findings. Additionally, as the ANDREAS instrument is still in development, its structure may be refined during the study. This presents potential risks such as construct drift or item overfitting, which will be mitigated through iterative item generation and expert review. Although proxy reports from parents and caregivers offer valuable insights, they may also introduce bias due to emotional, cultural, or interpretive factors. Furthermore, because the instrument is being developed within an Italian sociolinguistic and cultural context, future cross-cultural adaptation and validation will be necessary before the tool can be applied in other healthcare systems.

Despite these limitations, this study represents a critical step in developing an effective pain assessment tool for children with DS. Variability in pain perception and expression may influence the reliability of assessments, and the use of proxy reporting, while essential, introduces an element of subjectivity. Future research should expand the sample size and examine the tool’s validity across a range of clinical settings while also accounting for the environmental and social factors that influence pain perception in this population.

## Conclusions

The ANDREA-S instrument is designed to provide a tailored, multidimensional approach to assessing acute pain in children with DS and mild to moderate cognitive impairment. By combining an adapted face-based self-report scale with proxy reports of validated behavioral indicators from parents, nurses, and other healthcare professionals, the tool aims to improve the recognition and quantification of pain in this population. Following a thorough psychometric evaluation, the instrument is expected to demonstrate strong reliability and validity, supporting its use in both clinical and home settings. Ultimately, ANDREA-S seeks to advance more equitable, timely, and individualized pain management for this vulnerable pediatric group.
